# ecmtool: fast and memory-efficient enumeration of elementary conversion modes

**DOI:** 10.1093/bioinformatics/btad095

**Published:** 2023-02-21

**Authors:** Bianca Buchner, Tom J Clement, Daan H de Groot, Jürgen Zanghellini

**Affiliations:** acib GmbH, Austrian Centre of Industrial Biotechnology, 1190 Vienna, Austria; Systems Biology Lab, Vrije Universiteit, 1081HV Amsterdam, The Netherlands; Biozentrum, Swiss Institute of Bioinformatics, University of Basel, 4056 Basel, Switzerland; Department of Analytical Chemistry, University of Vienna, 1090 Vienna, Austria

## Abstract

**Motivation:**

Characterizing all steady-state flux distributions in metabolic models remains limited to small models due to the explosion of possibilities. Often it is sufficient to look only at all possible overall conversions a cell can catalyze ignoring the details of intracellular metabolism. Such a characterization is achieved by elementary conversion modes (ECMs), which can be conveniently computed with ecmtool. However, currently, ecmtool is memory intensive, and it cannot be aided appreciably by parallelization.

**Results:**

We integrate mplrs—a scalable parallel vertex enumeration method—into ecmtool. This speeds up computation, drastically reduces memory requirements and enables ecmtool’s use in standard and high-performance computing environments. We show the new capabilities by enumerating all feasible ECMs of the near-complete metabolic model of the minimal cell JCVI-syn3.0. Despite the cell’s minimal character, the model gives rise to $4.2\times 10^{9}$ ECMs and still contains several redundant sub-networks.

**Availability and implementation:**

ecmtool is available at https://github.com/SystemsBioinformatics/ecmtool.

**Supplementary information:**

Supplementary data are available at *Bioinformatics* online.

## 1 Introduction

Understanding the genotype–phenotype relation is key in systems biology. Constraint-based modeling is one method to approach this question. Given an organism-specific genome-scale metabolic model (GSMM), all steady-state capabilities of that organism can be understood as conic combinations of minimal pathways (Schuster and Hilgetag, 1994), known as elementary flux modes (EFMs). A full EFM analysis remains limited to core metabolic models (Buchner and Zanghellini, 2021). However, many EFMs show identical stoichiometric net conversions from nutrients to products. Thus, it should be easier to characterize ‘only’ an organism’s metabolic interaction with its environment than to determine how this is achieved. Elementary conversion modes (ECMs) achieve precisely that. ECMs form a minimal set of steady-state net conversions (Urbanczik and Wagner, 2005), whose conic combinations represent all feasible bio-transformations an organism can perform. Recently, we published ecmtool (Clement *et al.*, 2021), which enumerates ECMs in metabolic models. However, ecmtool’s current implementation is memory intensive, prohibiting large-scale networks’ analysis. We present an update to ecmtool that eliminates memory limitations and speeds up enumeration by parallel computation. To illustrate the new capabilities, we compute all ECMs in the GSMM of the synthetic minimal cell JCVI-syn3A growing on a complex medium.

## 2 Approach

ECMs are minimal generators of the space C of steady-state conversions given by the model’s stoichiometric matrix ***N*** (Clement *et al.*, 2021),


(1) $$C=\{\;ċ=\text{Nr}\;|\;c_{i}=0\;\text{for}\;i\in \mathrm{Int},r_{j}\geq 0\;\text{for}\;j\in \mathrm{Irrev}\;\}.$$

**Fig. 1.**
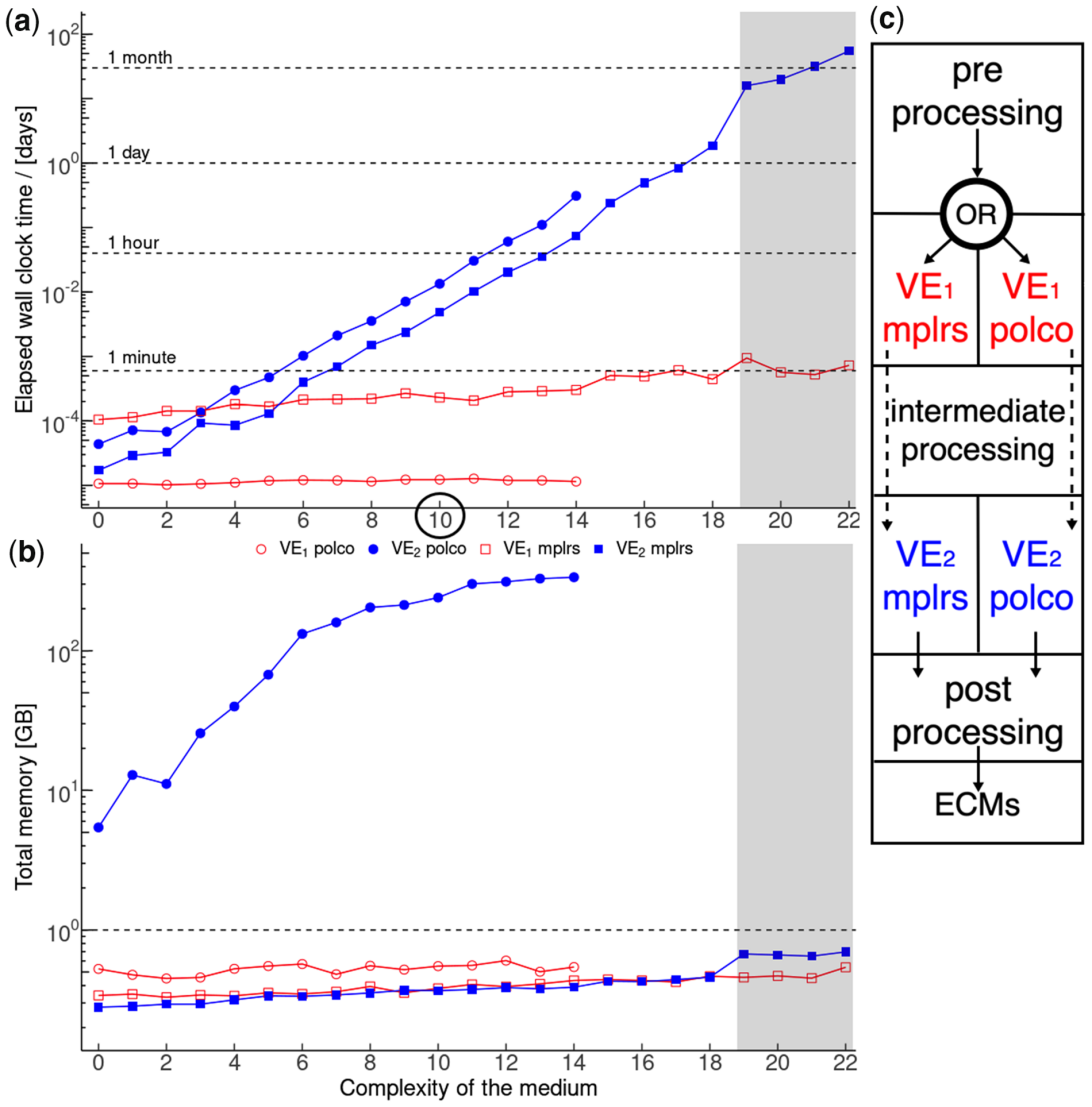
(**a**) Run time and (**b**) memory consumption of ecmtool using mplrs (squares) or polco (circles) as a function of the number of excess nutrients in the minimal medium of JCVI-syn3A. Empty and full symbols indicate VE_1_ and VE_2_, respectively; see (a). 20 and 60 (shaded area) threads were used. Shaded data are true values scaled with 60/20. polco’s java machine was restricted to 300 GB. For 15 medium components, ecmtool (polco) was aborted after 2 days without progress. (**c**) Structogram of ecmtool. The circle marks the model with 10 components used for a scaling study shown in Supplementary Figure S1

Here, the internal concentrations $c_{i},\;i\in \mathrm{Int}$ are in steady-state, and irreversible fluxes $r_{j}\geq 0$, j∈Irrev are non-negative. Finding the minimal set of generators of (1) can be reduced to twice solving a vertex/ray enumeration (VE) problem of a convex polyhedron (Clement *et al.*, 2021), see Figure 1c. For these steps, ecmtool uses a double-description algorithm implemented in polco (Terzer and Stelling, 2008). polco is memory intensive. It starts with a set of ‘candidate-ECMs’, which it then refines until the full set is found; the number of candidate-ECMs at intermediate steps can be huge and always needs to be kept in memory. We replaced polco with a parallelized, and memory-efficient lexicographic reverse search (LRS) implemented in mplrs (Avis and Jordan, 2018). LRS reverses the simplex method. It first finds a vertex/ray on the polyhedron, moves along the edges of the polyhedron and traces back all starting points that return that initial vertex in linear optimization. These starting points are the ECMs, and a unique path visits every ECM only once. Thus, no ECM needs to be stored, resulting in minimal memory requirements. Moreover, LRS can be split into independent, non-communicating sub-problems that can be efficiently parallelized.

ecmtool’s design is shown in Figure 1c. mplrs is the default, and users specify the number of threads via the command line.

## 3 Results and discussion

We enumerated all ECMs in a GSMM of the synthetic minimal cell JCVI-syn3A (Breuer *et al.*, 2019) with ecmtool. The model contains 304 metabolites, 338 reactions (incl. 69 exchange reactions) and 155 genes. Within 2.6 weeks, we enumerated 4 212 839 045 ECMs using 60 threads.

In Figure 1a, we compare the length of the two VE phases. With polco VE_1_ takes about 1 s independent of the medium’s complexity, while mplrs run time rises from 10 s to 1 min. However, for increasing model size the length of VE_2_ increases exponentially for both algorithms and quickly dominates total run time. During this phase, mplrs is on average 2.8× faster than polco. Moreover, with 15 extra medium components, mplrs finished within 5.7 h using 20 threads, while polco was terminated after 2 days as it did not finish within twice the expected run time. At this point, polco was severely limited by memory consumption reaching 300 GB, Figure 1b. mplrs, however, required far less than 1 GB even for the largest model with 22 excess medium components. With mplrs more threads reduce the run time further as it almost ideally scales with the number of threads; see Supplementary Figure S1. In contrast, polco is not designed for parallelization and thus does not benefit from adding more threads. Finally, we verified that mplrs and polco computed identical sets of ECMs. Thus, the updated ecmtool is faster, more resource conservative than its predecessor, and enables analysis at a previously infeasible scale.

Although there are differences in the spectra of byproducts that can be produced from individual nutrients (Supplementary Fig. S3), we found that the number of ECMs in JCVI-syn3A approximately doubles with each added nutrient (Supplementary Fig. S2). Such behavior is consistent with a bow-tie structure of metabolism, where a range of essentially independent nutrients fan in a highly redundant core (Csete and Doyle, 2004).

We computed all ECMs and EFMs in JCVI-syn3A growing on only an essential subset of the nutrients and verified that all projections of EFMs on the set of exchange reactions are either identical to an ECM (12.56% of all EFMs) or a conic combination of ECMs (87.44% of all EFMs).

In the former group, we find that every ECM gives the net conversion for on average 23 EFMs (Supplementary Fig. S4), which indicates high redundancy in the network. For example, 3426 ECMs encoded 20 EFMs each. These 20 EFMs were constructed from the combination of four ‘modules’ of two parallel enzymes plus one futile cycle coupled to each module ($2^{4}+1\times 4=20$, see Supplementary Fig. S5) in the nucleotide metabolism. Similar multiplicative growth in the number of EFMs due to metabolic modules has been previously observed (Kelk *et al.*, 2012).

The latter group indicates that in JCVI-syn3A, most EFMs represent stoichiometrically coupled production (or consumption) of (external) metabolites, which alone are produced (or consumed) more efficiently.

## 4 Conclusions

We presented an update to ecmtool that uses mplrs (Avis and Jordan, 2018) for parallelized ECM enumeration. The current version was three times faster and showed negligible memory consumption. ecmtool runs in any environment, from small computer clusters to high-performance computing systems, but benefits much more from large-scale parallelization than before. We enumerated all ECMs in the synthetic minimal cell JCVI-syn3.0 growing on complex media. ecmtool was able to characterize all metabolic interactions within 2.6 weeks using 60 threads. This scaling step paves the way to, e.g. unbiased study emerging properties of microbial metabolic interactions in (small) communities.

Financial Support: None declared.

*Conflict of Interest*: None declared.

## Supplementary Material

btad095_Supplementary_Data
