# Supplementary material for: ecmtool: fast and memory-efficient enumeration of elementary conversion modes
Source: Bioinformatics. 2023 Feb 21;39(3):btad095. doi: 10.1093/bioinformatics/btad095 (PMC9982354; doi:10.1093/bioinformatics/btad095)
Supplement: btad095_Supplementary_Data [file btad095_supplementary_data.pdf]

picture(0,0)(-35,0)(1,0)30 (0,35)(0,-1)30 picture picture(0,0)(35,0)(-1,0)30 (0,35)(0,-1)30 picture

Bioinformatics
doi.10.1093/bioinformatics/xxxxxx
Advance Access Publication Date: Monday 7th November, 2022
Applications Note

OXFORD

Supplementary material

`ecmtool`: fast and memory efficient enumeration of elementary conversion modes

Bianca Buchner [1], Tom Clement [2], Daan de Groot [3,*], and Jürgen Zanghellini [4,*]

[1] acib GmbH, Austrian Centre of Industrial Biotechnology, 1190 Vienna, Austria, EU,
[2] Systems Biology Lab, Vrije Universiteit, Amsterdam, 1081HV, The Netherlands,
[3] Biozentrum, University of Basel, and Swiss Institute of Bioinformatics, Basel, 4056, Switzerland and
[4] Department of Analytical Chemistry, University of Vienna, 1090 Vienna, Austria, EU.

* To whom correspondence should be addressed.

Associate Editor: XXXXXXX

Received on XXXXX; revised on XXXXX; accepted on XXXXX

Contact: daanhugodegroot@gmail.com, juergen.zanghellini@univie.ac.at

© The Author 2015. Published by Oxford University Press. All rights reserved. For permissions, please e-mail: journals.permissions@oup.com

picture(0,0)(-35,0)(1,0)30 (0,-35)(0,1)30 picture picture(0,0)(35,0)(-1,0)30 (0,-35)(0,1)30 picture

"output" — 2022/11/7 — page 1 — #1

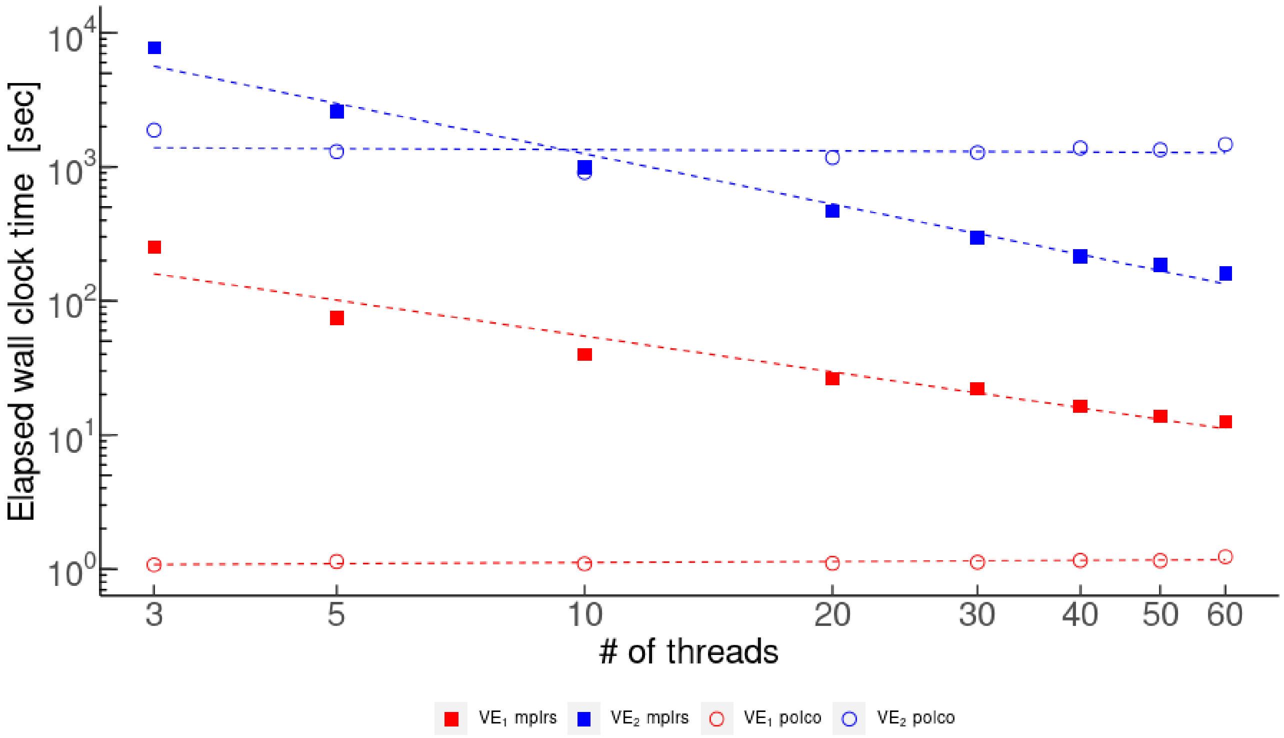

Fig. S1. Scaling behavior of `mplrs` (squares) and `polco` (circles) computing all vertices in vertex/ray enumeration (VE) phase VE_1 (red) and VE_2 (blue), respectively in a model with ten additional medium components as a function of the maximum number of available threads. Both axes are plotted on logarithmic scales. Trend lines indicate `polco`'s average wall time and a fit to `mplrs`' wall time, assuming ideal parallelization behavior, i.e. wall time $= C/(\text{\# of threads})$ with fitting parameter C.

picture(0,0)(-35,0)(1,0)30 (0,35)(0,-1)30 picture picture(0,0)(35,0)(-1,0)30 (0,35)(0,-1)30 picture

(a) (b)

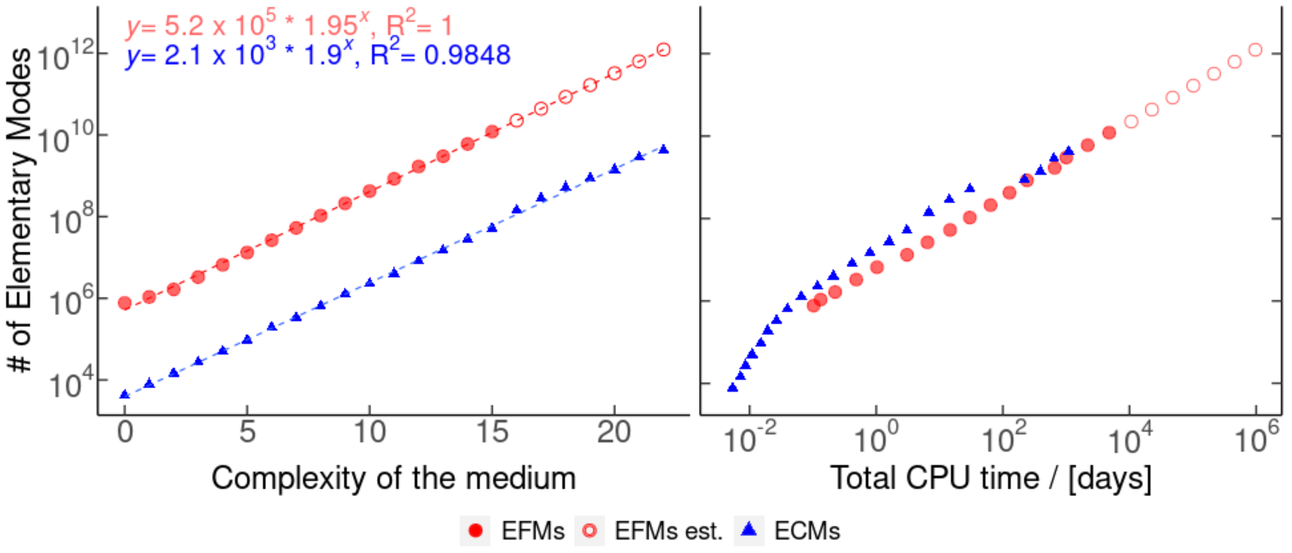

Fig. S2. Number of elementary conversion modes (ECMs) (triangles) and elementary flux modes (EFMs) (circles) as a function of the number of excess nutrients in the minimal medium of JCVI-syn3A. Full and open symbols represent measured and predicted values based on a fit to the measured data, respectively. The displayed number of ECMs corresponds to the mean of the ECMs from models with a fixed total number of medium components, but random composition, see Table S1. (b) Number of ECMs (triangles) and EFMs (circles) as a function of the total central processing unit (CPU) time averaged over all calculated instances, see Table S1. ECMs and EFMs were enumerated with the `mplrs` algorithm. `ecmtool` was used for ECM and `EFMlrs` (Buchner and Zanghellini, 2021) for EFM calculations [data taken from Buchner and Zanghellini (2021)]. Again empty circles represent estimates based on a fit to measured data (full circles).

Table S1. Set of models used to compute the ECMs in Fig. S2. For a fixed number of additional components in the medium (#components), we constructed a set of unique models (#calculated_models) of all possible models (#possible_models). For #components $\in \{0, 1, 2\}$ we constructed all possible models with different medium compositions. For #components $= 3$ and #components $\in \{4, 5\}$ we constructed $10\,\%$ and $1\,\%$ of all possible models, respectively, by randomly selecting their medium components. For #components ≥ 6 we constructed one model each by successively adding a so far unused medium component. In the set of calculated models, we counted the number of models (#maxECM_models and #minECM_models) with the maximum (max.#ECMs) and the minimum (min.#ECMs) number of ECMs, respectively. The last column, mean.#ECMs, is shown in Fig. S2 and denotes the arithmetic mean of the ECMs across the set of #calculated_models. Colored rows are further analyzed in Fig. S3.

#components	#possible_models	#calculated_models	#maxECM_models	#minECM_models	max.#ECMs	min.#ECM	mean #ECMs
0	1	1	1	1	4,132	4,132	4,132
1	22	22	15	4	8,254	6,178	7,774
2	231	231	95	1	16,498	9,232	14,541
3	1,540	154	43	1	32,986	16,564	27,166
4	7,315	74	9	1	65,962	27,774	50,456
5	26,334	264	21	1	131,914	52,026	94,202
6	74,613	50	4	1	263,818	116,458	191,037
7	170,544	50	1	1	527,626	178,650	337,106
8	319,770	50	1	1	947,722	404,586	640,212
9	497,420	50	2	1	2,110,474	715,530	1,274,593
10	646,646	50	1	1	3,377,162	1,019,210	2,254,895
11	705,432	50	1	1	6,754,314	2,422,346	4,035,214
12	646,646	50	1	1	14,876,682	4,076,810	8,079,122
13	497,420	50	1	2	24,928,266	8,960,010	14,821,473
14	319,770	50	1	3	45,367,306	16,307,210	27,908,690
15	170,544	50	1	2	73,953,438	32,000,010	51,180,496
16	74,613	1	1	1	142,049,290	142,049,290	142,049,290
17	26,334	1	1	1	284,098,570	284,098,570	284,098,570
18	7,315	1	1	1	514,523,146	514,523,146	514,523,146
19	1,540	1	1	1	869,854,722	869,854,722	869,854,722
20	231	1	1	1	1,403,221,121	1,403,221,121	1,403,221,121
21	22	1	1	1	2,821,027,740	2,821,027,740	2,821,027,740
22	1	1	1	1	4,212,839,045	4,212,839,045	4,212,839,045

picture(0,0)(-35,0)(1,0)30 (0,-35)(0,1)30 picture picture(0,0)(35,0)(-1,0)30 (0,-35)(0,1)30 picture

“output” — 2022/11/7 — page 3 — #3

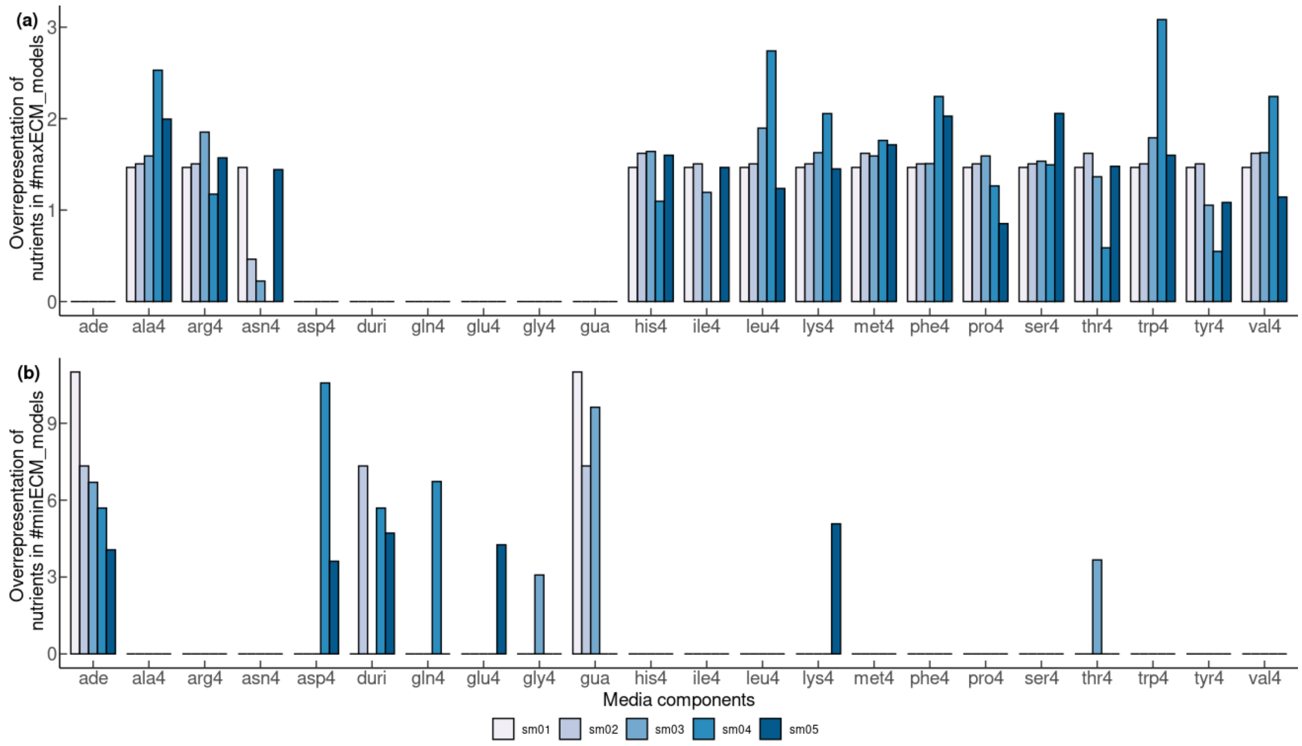

Fig. S3. (Over-)representation of medium components in the set of models with (a) maximum number of ECMs (#maxECM_models), and (b) minimum number of ECMs (#minECM_models), see Table S1 for details on the construction of these sets of models. For a fixed number of additional medium components, over-representation is measured as the ratio between the fractional occurrence of a medium component in the set of models with a maximum (or minimum) number of ECMs to the fractional occurrence of a medium component in all models. When a medium component is strongly over-represented in the models with a maximal number of ECMs, this indicates that this component is used in many different conversions, and thus facilitates a large metabolic diversity. Note that the medium components adenine (ade), aspartate (asp4), deoxyuridine (duri), glutamine (gln4), glutamate (glu4), glycine (gly4), and guanine (gua) were never found in the set of models with the largest numbers of ECMs, while they are over-represented in the set of models with the smallest number of ECMs.

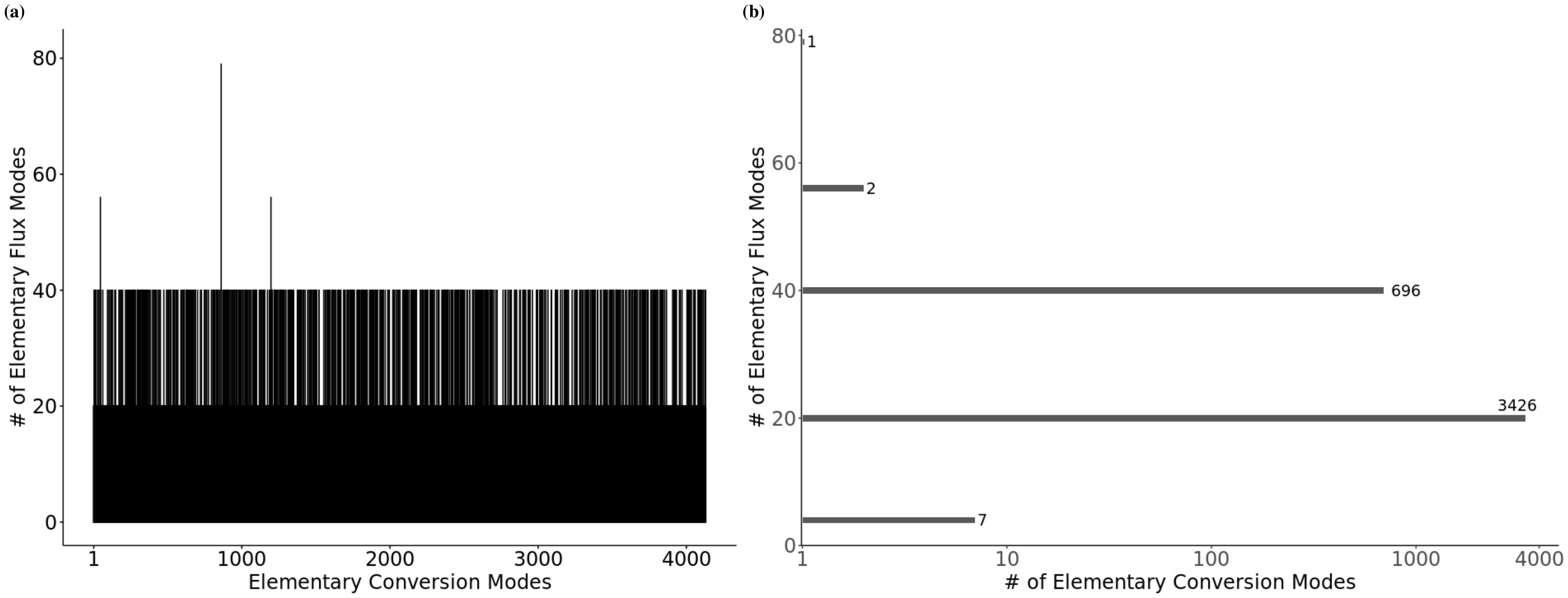

Fig. S4. Count of EFMs per ECM (panel a) and count of EFMs per ECM count (panel b). The x axis of the plot in panel b is in logarithmic scale. In total 96,579 EFMs project directly onto 4,132 ECMs. 83% of ECMs represent 20 EFMs each (see the longest bar at $y = 20$ in panel b). On average each ECM codes for 23 EFMs. However, one ECM was found that accounts for 79 EFMs. In panel (a), this is shown by the long spike, and in panel (b) by the short bar at $y = 79$.

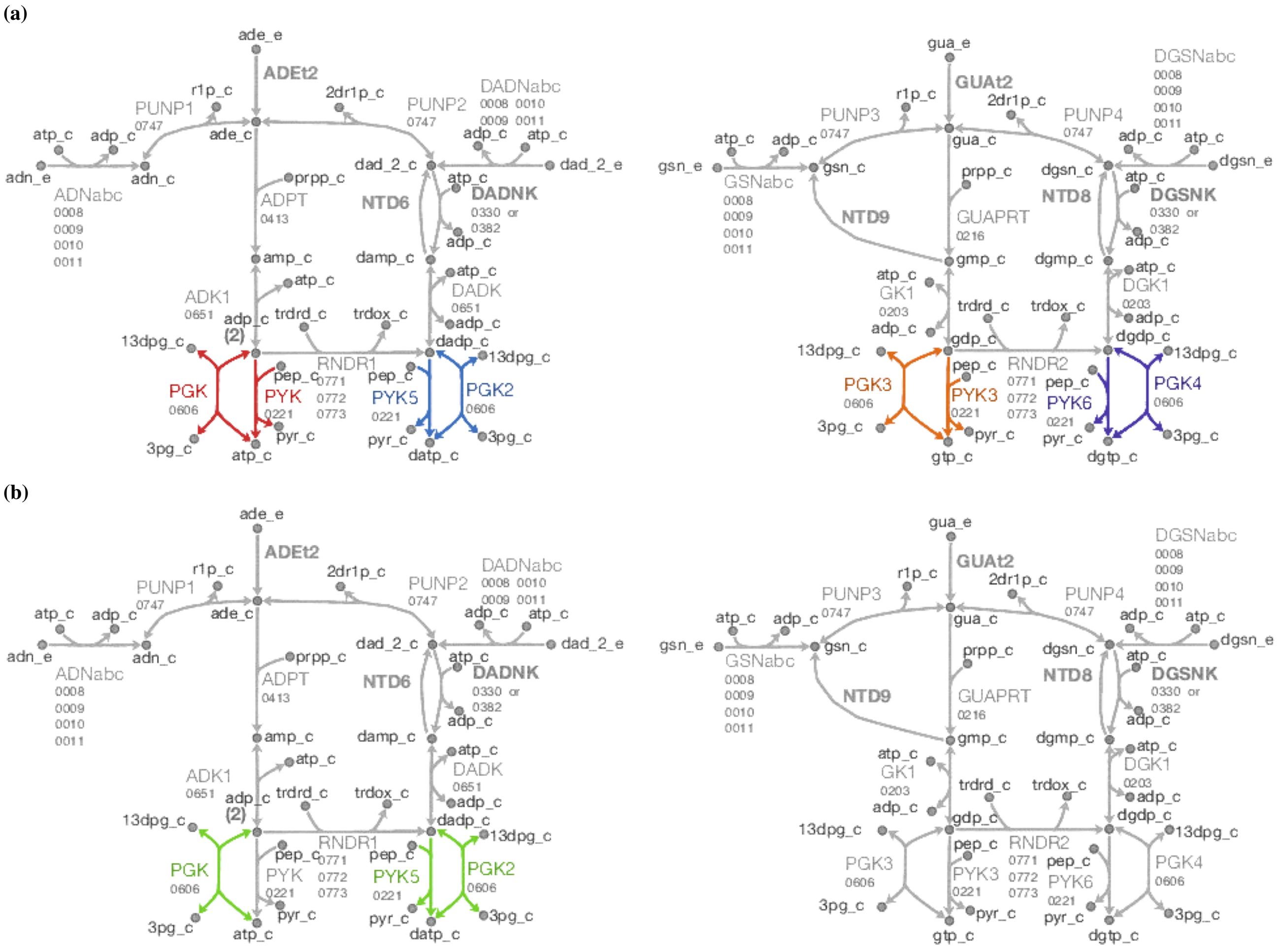

Fig. S5. Panel a, four metabolic sub-networks (indicated in red, blue, orange, and purple) consisting of two alternative enzymes (phosphoglycerate kinase, PKG; and Pyruvate kinase, PYK) each in the nucleotide metabolism of JCVI-syn3A. Panel b, exemplary coupling of two sub-networks (see panel a, red and blue sub-networks) via a futile cycle with the isozymes PGK and PGK2. Four of such futile cycle couplings with PGK can occur, PYK–PGK, PYK5–PGK2–PGK (indicated in green in panel b), PYK3–PGK3–PGK, and PYK6–PGK4–PYK. In total the combination of these five patterns give rise to $2^4 + 1 \times 4 = 20$ different EFMs that map onto one of the 3426 ECMs in Fig. S4. The pathway map was taken from Breuer et al. (2019) and modified.

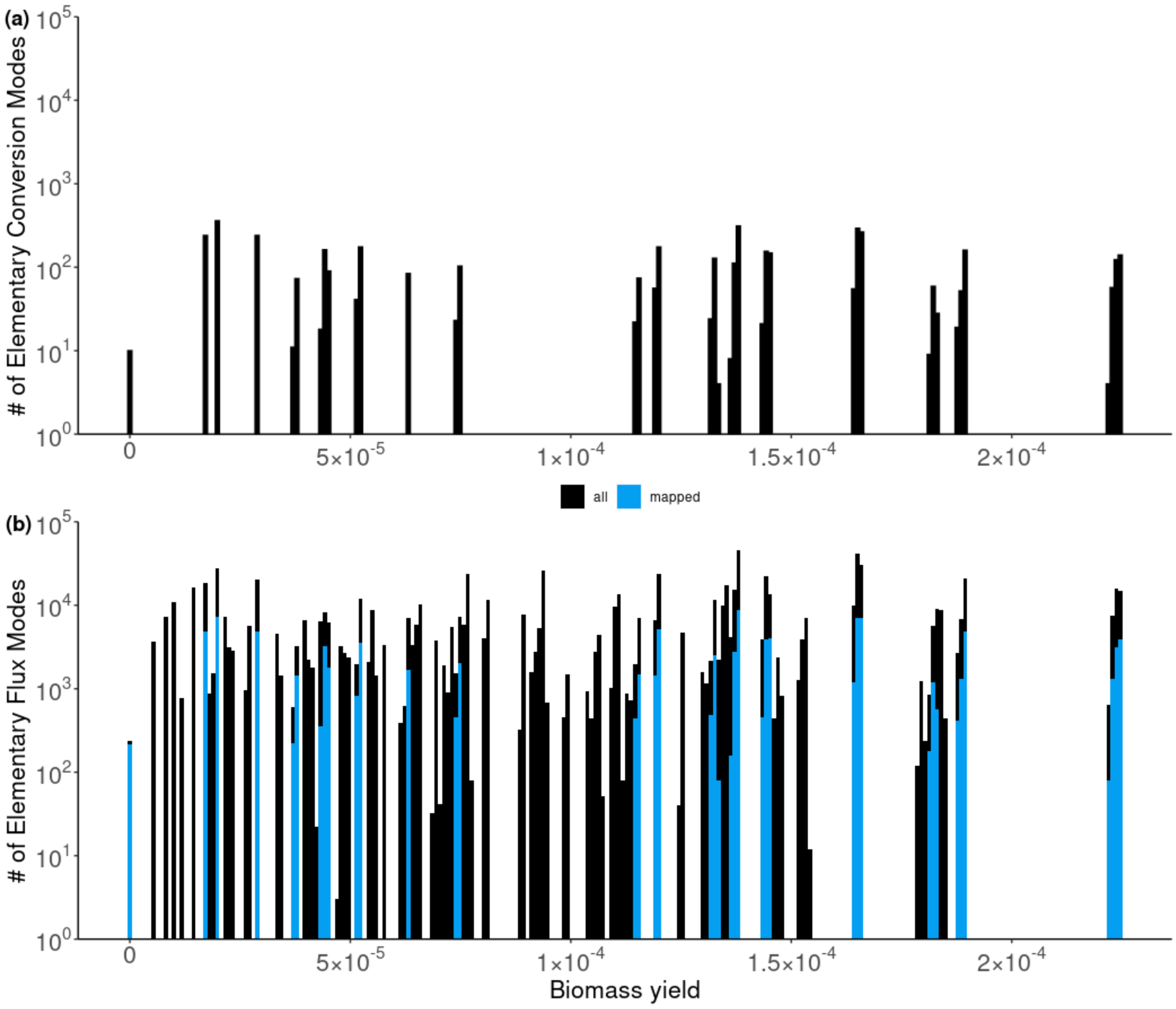

Fig. S6. Histograms of ECMs and EFMs. ECM count (panel a) and EFM count (panel b) as a function of the respective biomass yield. Blue bars in panel b show the number of EFMs whose projection is identical to an ECM, and black bars show the number of all EFMs at that yield. The highest ECM count occurs at a yield of 1.65×10^{-4}. It is produced by 444 ECMs corresponding to 11,280 (blue) and 58,440 (black) EFMs, respectively. 10 ECMs – or 219 (blue) and 234 (black) EFMs, respectively – do not produce any biomass. The highest yield is 2.24×10^{-4}. It is obtained by 156 ECMs or 4,240 (blue) and 17,440 (black) EFMs, respectively.

References

Breuer, M., Earnest, T. M., Merryman, C., Wise, K. S., Sun, L., Lynott, M. R., Hutchison, C. A., Smith, H. O., Lapek, J. D., Gonzalez, D. J., de Crécy-Lagard, V., Haas, D., Hanson, A. D., Labhsetwar, P., Glass, J. I., and Luthey-Schulten, Z. (2019). Essential metabolism for a minimal cell. *eLife*, **8**, e36842.

Buchner, B. and Zanghellini, J. (2021). EFMlrs: a Python package for EFM enumeration via lexicographic reverse search. *BMC Bioinformatics*, **22**(1), 547.

picture(0,0)(-35,0)(1,0)30 (0,35)(0,-1)30 picture picture(0,0)(35,0)(-1,0)30 (0,35)(0,-1)30 picture

Table S2. Author's contribution

Contributor roles	BAB	TC	DHdG	JZ
Conceptualization			■	■
Data curation	■			
Formal analysis	■			
Funding acquisition				■
Investigation	■			
Methodology	■	■	■	■
Project administration				■
Resources	■	■	■	■
Software	■	■	■	
Supervision				■
Validation	■			
Visualization	■			■
Writing – original draft				■
Writing – review & editing	■	■	■	■

picture(0,0)(-35,0)(1,0)30 (0,-35)(0,1)30 picture picture(0,0)(35,0)(-1,0)30 (0,-35)(0,1)30 picture

"output" — 2022/11/7 — page 8 — #8
